# Olden-Time Artificial Teeth

**Published:** 1891-02

**Authors:** 


ARTICLE IV.

### OLDEN-TIME ARTIFICIAL TEETH.

A curious old book has just come into our hands, kindly lent by Mr. Rutterford, which already in the year 1816 had reached its fifth edition. It is entitled "A Dissertation on Artificial Teeth," by M. de Chemant. Apparently its object was to act as a kind of advertisement for M. De Chemant, since his address is printed in a very prominent manner; indeed, he devotes a page to informing those persons who may desire to consult him on the subject of his mineral paste teeth, that he would be obliged to them if they would, on the preceding day, making their appointment, etc., etc.—We fancy it would be a case of the "pot calling the kettle black," were we, of this generation, to find fault with this comely old gentleman (his portrait is given) for so doing. Unfortunately, it is now-a days more the rule than the exception for those who rush into print, to let their address occupy a prominent position in the book, though perhaps they do not so openly (should we say honestly?) let the real why and wherefore of the book's appearance be seen.

The chief point of interest in the book centres in a sheet of engravings illustrating the various types of dentures which M. De Chemant was prepared to supply to his patients. Here is a porcelain bridge of ten teeth supported by four pivots by which they are fastened to the stumps remaining in the jaw. A single tooth for all the world like a Logan Crown. A single tooth to be fixed by means of a small plate of gold, or, as we in this inventive (sic) age would say, a bridge for one tooth. A row of teeth to be supported by ligatures; this idea we would suggest to some of those inventive genii, who are hard up for something to patent. We do not for a moment wish to detract from the merit due to those who work out original ideas, because some one else has done it all before. But does it not seem a waste of energy? Whether the fact be due to lack of reading on the part of the re-inventor or to our librarians letting their collection of books be but a miscellaneous heap of curiosities, not a classified collection with a general index and guide, we do not know. We remember reading somewhere, that in one of the German Universities there is a general index to the whole library, from which a man can see at a glance what has been done in each branch of study, so that it is possible to continue a line of research from the point where the last man left off, without wasting time going over the old ground.

Very interesting it is to be brought once again in contact with the famous medical men of the beginning of the century. Here we meet John Hunter, who introduces De Chemant to one of his patients, and these six cases of transplantation, followed by "venereal disease" which led him to give up "this cruel practice," are mentioned. Here again, too, the "immortal Jenner," in whose presence and for one of whose patients he removed fifteen or seventeen stumps, decayed even unto the socket. Here, too, Sir Walter Farquhar, M. Vicq D'Azyr and others.

M. De Chemant speake of these teeth as his invention, and thus records the circumstances, "to satisfy the curiosity of the reader":

In 1788, when I exercised the profession of a surgeon, I was consulted by a lady who had fallen into such a state of weakness as produced considerable fears of her life. On approaching her I perceived a tainted odor, which I thought proceeded from her lungs or her teeth, which were black. I examined her mouth, and was struck with the bad state of a set of human teeth implanted on a base of the tooth of the hippopotamus. This set of teeth being removed, I perceived her mouth to be almost entirely covered with small ulcers, and I had no doubt but that her disease was the effect of the putrid exhalations which proceeded from the set of teeth, and which corrupted the air she breathed; what confirmed this conjecture was that, after having laid these teeth aside, her health improved in a few days. Perceiving that this lady would not do without artificial teeth, I advised her to have several sets of teeth at the same time, so that she might change them often, after having washed them and let them dry. She did so, and her health became perfectly re-established in the course of some months.

"But as teeth of this kind require to be renewed frequently, they occasion a very great expense, and even, notwithstanding their frequent renewal, they always produce a bad smell. I was induced from that time to reflect on the possibility and the means of making teeth and sets of teeth of durable and incorruptible materials. I examined almost all the substances of the mineral kingdom, and at length composed a paste, which, when it is baked, has every desirable advantage."

Now, as a matter of fact, or rather, according to Piggot's "Dental Chemistry," they were discovered by an apothecary of St. Germain, Duchateau by name. He wore artificial dentures of ivory and natural teeth, but found they rapidly became tainted by the various disagreeable odors emanating from his wares, the porous animal substances becoming rapidly impregnated by the effluvia. Mr. Guerard undertook the manufacture in 1776. Sets were made for various distinguished personages, but he failed from want of knowledge in the practical duties of a dentist. In 1788, Dubois Chemant bought the right, and managed to attract the attention of the French Academy, who appointed a sub-committee to examine the teeth. This committee found various imperfections in the teeth, a fact which, by-the-by, M. de Chemant does *not* record in his book, and one of its members, Dubois Foucou, improved them very much. Practically, little alteration has been made in this substance even in modern times.—*British Journal of Dental Science.*

